# IgM as a novel predictor of disease progression in secondary focal segmental glomerulosclerosis

**DOI:** 10.3325/cmj.2017.58.281

**Published:** 2017-08

**Authors:** Arijana Pačić, Petar Šenjug, Jasna Bacalja, Miroslav Tišljar, Ivica Horvatić, Stela Bulimbašić, Mladen Knotek, Krešimir Galešić, Danica Galešić, Ljubanović

**Affiliations:** 1 Department of Pathology and Cytology, Dubrava University Hospital, Zagreb, Croatia; 2 Department of Nephrology, Dubrava University Hospital, Zagreb, Croatia; 3 Department of Pathology and Cytology, Zagreb University Hospital Centre, Zagreb, Croatia; 4 Department of Nephrology, Merkur University Hospital, Zagreb, Croatia; 5 University of Zagreb, School of Medicine, Zagreb, Croatia

## Abstract

**Aim:**

To determine the role of immunoglobulin M (IgM) deposits in clinical manifestations, disease outcome, and treatment response of idiopathic and secondary focal segmental glomerulosclerosis (FSGS).

**Methods:**

Kidney biopsy specimens of 171 patients diagnosed with FSGS (primary and secondary) and 50 control patients were retrospectively included in the study. For each patient, clinical and outcome data were obtained and compared to morphological parameters, including immunofluorescence analysis of mesangial IgM and complement 3 (C3) deposits analyzed on kidney biopsy samples.

**Results:**

There were significant positive correlations between IgM and C3 deposition in secondary FSGS (*P* < 0.001) and between IgM and mesangial deposits detected by electron microscopy in secondary FSGS (*P* = 0.015), which indicated that higher IgM deposition correlated with higher C3 deposition and mesangial deposits only in secondary FSGS. Patients with secondary FSGS and the deposition of IgM showed inferior renal outcomes at earlier time points in comparison with patients with negative IgM expression (*P* = 0.022).

**Conclusions:**

We detected a positive correlation between IgM and C3 in secondary FSGS. The association between IgM deposition and worse renal outcome in secondary FSGS indicates that IgM may play a role in the progression of this disease.

Focal segmental glomerulosclerosis (FSGS) is a clinicopathological syndrome that usually manifests clinically as nephrotic syndrome and morphologically as focal and segmental glomerular sclerosis under light microscopy (LM), a foot-process effacement under electron microscopy (EM), and occasional immunoglobulin M (IgM) deposits in immunofluorescent (IF) analysis (1-5). Both primary (idiopathic) and secondary forms of this syndrome have been described, with five diverse morphological types that occur in both clinical forms (1,6). Secondary FSGS has diverse etiology including gene mutations, viruses, toxins, and structural and functional adaptation, such as hypertrophy, hyperfiltration, and loss of renal mass (7).

In addition to FSGS, mesangial deposits of IgM can be found in various primary and secondary renal diseases, such as minimal change disease, mesangioproliferative glomerulonephritis, hypertensive nephrosclerosis, and diabetic nephropathy (8-12). The presence of glomerular IgM deposits in FSGS is interpreted as passive entrapment of the large IgM molecule within sclerotic areas. However, diffuse granular IgM deposits are also present in FSGS in non-sclerotic mesangial areas. Furthermore, IgM is frequently accompanied by C3 and C4 deposits (10,13,14). In comparison with sclerotic areas, mesangial staining for IgM and C3 in non-sclerotic segments is less intense (15).

Recently, the presence of IgM in non-sclerotic areas of glomeruli has been explained by specific natural IgM binding to neoantigens exposed in injured or stressed glomeruli (16). The recent experimental study using an animal model of glomerulosclerosis documented IgM-mediated activation of the complement system and its role in FSGS progression, a finding corroborated in human biopsies from patients with idiopathic FSGS (17). A large retrospective study focusing exclusively on patients with primary FSGS and mostly on sclerotic lesions further highlighted the correlation between IgM and C3 glomerular deposits and unfavorable therapeutic responses and worse renal outcomes (18).

The aim of our study was to investigate the potential correlation between IgM and C3 mesangial deposits in kidney biopsy tissue of patients with both primary and secondary FSGS. The hypothesis tested was that the presence of IgM deposits in non-sclerotic areas may predict disease progression in some patients irrespective of disease etiology.

## METHODS

### Patients

We retrospectively analyzed kidney biopsy specimens and clinical data from diagnostic biopsies obtained between 2003 and 2014 at the Department of Pathology and Cytology of Dubrava University Hospital in Zagreb, Croatia, including kidney biopsy samples of 171 adult patients with biopsy-proven FSGS and 50 control subjects consisting of consecutive patients diagnosed with either thin glomerular basement nephropathy or without significant changes on renal biopsy specimen. The diagnosis of FSGS was made according to the FSGS definition in the Columbia classification (1). Patients diagnosed with FSGS were clinically divided in those with primary and those with secondary FSGS. Patients without clinical evidence for secondary FSGS, such as obesity, reflux nephropathy, positive family history, unilateral kidney, and hypertension, were considered as having primary FSGS. Of 171 FSGS patients, for 10 patients there were insufficient clinical data to adequately classify FSGS as primary or secondary, for 2 patients there were insufficient kidney biopsy specimens for adequate IF analysis (no IgM and C3 deposition data), and for 3 patients both information was missing. Thus, the combined analysis of primary and secondary FSGS with IF parameters included 166 patients, and the divided analysis of primary and secondary FSGS with IF parameters included 156 patients.

### Data collection, treatment, and outcome

For each patient included in the study, available clinical data before kidney biopsy were collected by reviewing the patients’ medical histories. The data collected included body mass index (BMI), cholesterol level, triglycerides level, plasma protein level, blood pressure, plasma IgM level, creatinine level, creatinine clearance, 24-hour proteinuria level, and serum albumin level. Clinical data during treatments and follow-up were collected from medical records. Comparison analyses were performed on available collected data.

Estimated glomerular filtration rate (eGFR) was expressed in mL/min/1.73 m^2^ and calculated according to the Chronic Kidney Disease Epidemiology Collaboration (CKD-EPI) formula (19).

Standard therapy for patients with primary FSGS consisted of a corticosteroid with immunosuppressive agents including cyclophosphamide, cyclosporine A, and tacrolimus and combined with renin-angiotensin-aldosterone system (RAAS) blockade. Patients admitted after 2012 received treatments according to the Kidney Disease Improving Global Outcomes (KDIGO) guidelines (20). Standard therapy for patients with secondary FSGS was solely RAAS blockade with either angiotensin-converting enzyme inhibitors or angiotensin receptor blockers.

For evaluation of treatment response, a complete remission was defined as proteinuria <0.3 g/24 h and stable or improved serum creatinine, while partial remission was defined as proteinuria 0.3-3.5 g/24 h and stable or improved serum creatinine. Treatment failure was defined as not reaching the criteria of complete or partial remission.

In terms of clinical outcome, we evaluated renal progression of the disease that was defined as permanent increase in serum creatinine by ≥50% and/or end stage renal disease and/or need for renal replacement therapy/dialysis, transplant or death (composite renal outcome).

### Renal histopathology

Renal biopsy was performed in all patients at the time of diagnosis. Renal specimens were evaluated using LM, IF, and EM. The material for LM was cut into serial sections. Each section was alternately stained with hematoxylin and eosin, periodic acid-Schiff, Masson’s trichrome, and Jones’ stain. Immunofluorescence was made on a frozen sample, and serial sections for direct IF were stained with antibodies against IgG, IgA, IgM, C3, C1q, fibrinogen, albumin, and the kappa and lambda light chains (Dako, Glostrup, Denmark). The tissues for EM were processed using standard methods (fixation in McDowell's fixative followed by 2% osmium tetroxide, contrasting 3% uranyl acetate, acetone dehydration, submersion in epoxy resin, and cutting ultrathin sections on a ultramicrotome) and examined using a JEOL 1400 (Jeol, Tokyo, Japan) electron microscope.

The histological type of FSGS, total number of glomeruli, number of glomeruli with global and segmental sclerosis, presence, quantity and distribution of IgM and C3 deposits, foot process effacement, degree of interstitial fibrosis and tubular atrophy, arteriolar hyalinosis, and arterial intimal thickening were determined. The histological type of FSGS was defined according to the criteria established by a group at Columbia University (1). The degree of interstitial fibrosis and tubular atrophy was determined semiquantitatively on a section stained with Masson’s trichrome and expressed as a percentage of renal cortex with interstitial fibrosis and tubular atrophy compared to the total area of cortex parenchyma. Arterial intimal thickening and arteriolar hyalinosis were defined according to the Banff classification (21). The degree of podocyte foot process effacement was determined using at least 10 EM photographs (magnification x8000) of randomly photographed regions of the glomeruli and expressed as a percentage of the glomerular basement membrane with podocyte foot process effacement compared to the total analyzed area of the glomerular basement membrane.

**Figure 1:**
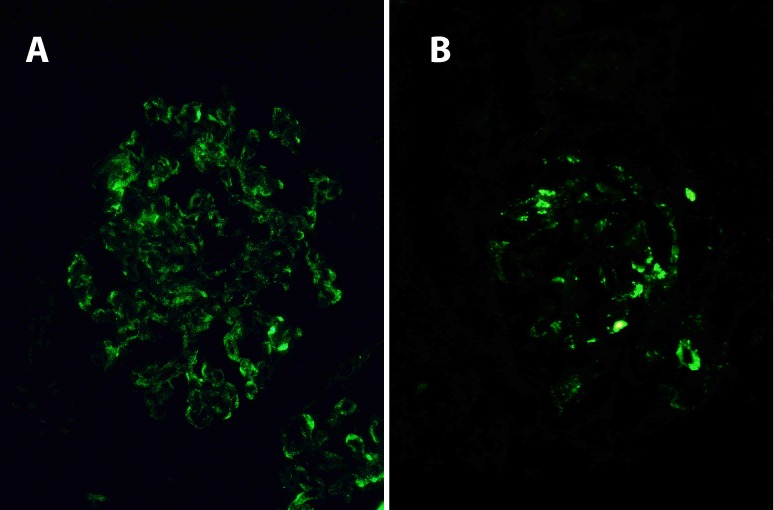
Glomerular deposits by direct immunofluorescence microscopy (x400). **A.** Diffuse granular staining (++) in the glomerulus for immunoglobulin M. **B.** Diffuse granular staining (+) in the glomerulus for complement 3.

Polyclonal rabbit anti-human IgM/ fluorescein isothiocyanate (FITC) and Dako FITC-conjugated rabbit anti-human C3c complement antibodies (Dako) were used according to the manufacturer’s directions. Positivity for IgM and C3 was determined semiquantitatively in snap-frozen sections. Only diffuse, global distribution of IgM and C3 in nonsclerotic areas was considered positive (Figure 1), and the intensity of the staining was graded as negative (0), weakly positive (+), moderately positive (++), and strongly positive (+++). Microscopic analyses were performed by two experienced nephropathologists.

### Statistical analysis

The normality of the data distribution was assessed with the Kolmogorov-Smirnov test and appropriate non-parametric tests were used in additional analyses. Differences between the FSGS and control groups and between the primary and secondary FSGS groups were analyzed with a χ^2^ test (categorical values) and a Mann-Whitney U test (quantitative values). Spearman correlation coefficients were used to analyze associations between IgM, C3 levels, and EM mesangial deposits in primary and secondary FSGS. Kaplan-Meier survival curves were used to study prognostic relevance, and corresponding log-rank (Mantel-Cox) test of equality for survival distributions was used to analyze different levels of IgM and C3 in primary and secondary FSGS over time for renal outcomes and response to treatment. Binary logistic regression was performed to analyze the impact of the number of different predictors on the likelihood of disease progression or complete or partial remission. Results were expressed as odds ratios (OR) with 95% confidence intervals (95% CI). If the *P* value of the candidate predictor in univariate survival analysis was <0.05, the predictor was included in the multivariable regression model. All statistical analyses were two tailed, and a *P* value <0.05 was considered as statistically significant. The data analysis software system IBM SPSS Statistics, version 21.0 (IBM Corporation Armonk, NY, USA), was used for statistical analysis.

## RESULTS

### Demographic, clinical, and histological characteristics

Of a total of 221 patients included in the study, 50 patients were in the control group and 171 in the FSGS group. Male gender was predominant in the combined (primary and secondary) FSGS patient group (Table 1). Additionally, the patient group was significantly older and had higher systolic and diastolic blood pressure and BMI values.

**Table 1:** Differences in characteristics between patients in the control group and combined focal segmental glomerulosclerosis group*

Characteristic	No. (%) of patients		*P*
	control group (n = 50)	FSGS group (n = 171)	
IgM			0.002^†^
negative	35 (70.0)	74 (44.6)	
positive	15 (30.0)	92 (55.4)	
C3			
negative	48 (96.0)	141 (84.9)	0.038^†^
positive	2 (4.0)	25 (15.1)	
IgM deposition			
IgM-, C3	35 (70.0)	74 (44.6)	0.005^†^
IgM+, C3-	13 (26.0)	69 (41.6)	
IgM+, C3+	2 (4.0)	23 (13.9)	
Mesangial deposits by EM^‡^			
negative	44 (88.0)	130 (82.8)	0.382^†^
positive	6 (12.0)	27 (17.2)	
Gender			
female	33 (66.0)	56 (32.7)	<0.001^†^
male	17 (34.0)	115 (67.3)	
Age (years; median, IQR)	39.0 (28.3-49.3)	50.0 (36.0-61.0)	<0.001^§^
Blood pressure (mmHg; median, IQR)			
systolic	120.0 (115.0-135.0)	145.0 (130.0-160.0)	0.006^§^
diastolic	77.5 (70.0-81.3)	90.0 (80.0-100.0)	<0.001^§^
BMI (kg/m^2^; median, IQR)	25.5 (23.5-30.0)	28.0 (24.9-32.1)	<0.001^†^

*Abbreviations: FSGS – focal segmental glomerulosclerosis, Ig – immunoglobulin; C – complement, EM – electron microscopy, IQR – interquartile range, BMI – body mass index.

†χ^2^ test.

‡Excluded patients without EM data for glomeruli analysis.

§Mann-Whitney *U* test.

A comparison between the primary and secondary FSGS groups showed that the secondary FSGS group had significantly higher values of BMI, systolic blood pressure, total serum protein, albumin, serum creatinine, and number of globally sclerotic glomeruli (Table 2). Serum cholesterol, creatinine clearance, proteinuria, total number of glomeruli, and the number of glomeruli with segmental sclerosis were significantly higher in the primary FSGS group. C3 distribution showed more prevalent expression in the secondary FSGS, podocyte foot process effacement showed a higher rate in primary FSGS, and arteriolar hyalinosis was higher in secondary FSGS (Table 3). Primary FSGS group had significantly higher proteinuria levels and immunosuppressive therapy rates.

**Table 2:** Differences between primary and secondary focal segmental glomerulosclerosis in quantitative clinical and morphological characteristics*

Parameter	Normal value range^†^	Primary FSGS (n = 47)			Secondary FSGS (n = 109)			*P^‡^*
		25th	50th (median)	75th	25th	50th (median)	75th	
Age (years)		32.0	48.0	63.0	38.0	50.5	60.0	0.769
BMI (kg/m^2^)		22.6	26.0	29.9	25.4	30.0	33.8	0.001
Systolic blood pressure (mmHg)		120.0	132.5	160.0	140.0	150.0	160.0	0.022
Diastolic blood pressure (mmHg)		80.0	90.0	100.0	80.0	90.0	100.0	0.151
Serum cholesterol (mmol/L)	<5	5.9	7.9	10.3	5.0	5.9	7.1	<0.001
Serum triglycerides (mmol/L)	<1.7	1.9	2.7	3.5	1.6	2.6	3.9	0.653
Total serum proteins (g/L)	60-78	41.0	54.0	61.0	62.7	69.0	74.8	<0.001
Albumin (g/L)	41-51	16.0	24.0	32.0	34.6	39.8	43.3	<0.001
Serum creatinine (µmol/L)	64-104	78.5	95.0	174.0	93.0	130.0	201.0	0.045
Creatinine clearance (ml/ min)^§^		60.5	90.5	109.4	42.0	70.0	101.6	0.040
Proteinuria (g/L)	<0.25	3.3	9.6	12.5	1.5	2.9	5.1	<0.001
eGFR (ml/min/1.73 m^2^)	>90	35.0	73.30	93.50	28.90	49.30	80.90	0.067
Total number of glomeruli		13.0	18.0	27.0	10.0	13.0	19.5	0.001
Number of globally sclerotic glomeruli		1.0	1.0	3.0	1.0	3.0	5.0	0.007
Number of glomeruli with segmental sclerosis		2.0	3.0	5.0	1.0	2.0	4.0	0.015
Time to response to treatment (months)		6.0	18.0	48.0	6.0	12.0	36.0	0.443
Time to combined renal outcome (months)		12.0	30.0	72.0	12.0	24.0	60.0	0.845

*Abbreviations: FSGS – focal segmental glomerulosclerosis, BMI – body mass index, eGFR –estimated glomerular filtration rate.

†Normal values of laboratory parameters at Department of Laboratory Diagnostics, Dubrava University Hospital, Zagreb.

‡Mann-Whitney *U* test.

§Estimate by Cockcroft-Gault formula.

**Table 3:** Differences between primary and secondary focal segmental glomerulosclerosis in morphological and clinical categorical characteristics*

Characteristics	No. (%) of patients		*P^†^*
	primary FSGS (n = 47)	secondary FSGS (n = 109)	
IgM deposition			0.321
negative	23 (48.9)	44 (40.4)	
positive	24 (51.1)	65 (59.7)	
C3 deposition			
negative	44 (93.6)	88 (80.7)	0.041
positive	3 (6.4)	21 (19.3)	
IgM and C3 deposition			
IgM-, C3-	23 (48.9)	44 (40.4)	0.067
IgM+, C3-	22 (46.8)	45 (41.3)	
IgM+, C3+	2 (4.3)	20 (18.4)	
C1q deposition			
negative	44 (93.6)	104 (95.4)	
positive	3 (6.4)	5 (4.6)	
IgA deposition			
negative	44 (93.6)	103 (94.5)	
positive	3 (6.4)	6 (5.5)	
IgG deposition			
negative	47 (100.0)	109 (100.0)	
Mesangial deposits by electron microscopy‡			
negative	34 (77.2)	88 (84.6)	0.283
positive	10 (22.7)	16 (15.4)	
Podocyte foot processes effacement‡			
podocyte foot preserved	1 (2.3)	20 (19.6)	<0.001
≤25% loss	4 (9.1)	27 (26.5)	
26%-50% loss	8 (18.2)	28 (27.5)	
≥50% loss	31 (70.5)	27 (26.5)	
FSGS type			
classic	16 (34.0)	61 (56.0)	<0.001
perihilar	5 (10.6)	44 (40.4)	
cellular	11 (23.4)	3 (2.8)	
tip	14 (29.8)	1 (0.9)	
collapsing	1 (2.1)	0 (0.0)	
Arterial intimal thickening			
no intimal thickening	28 (59.6)	46 (42.2)	0.106
≤25% lumen constriction	6 (12.8)	15 (13.8)	
26%-50% lumen constriction	10 (21.3)	26 (23.9)	
≥50% lumen constriction	3 (6.4)	22 (20.2)	
Arteriolar hyalinosis			
without hyalinosis	29 (61.7)	28 (25.7)	<0.001
nodular hyalinosis of one arteriole	5 (10.6)	14 (12.8)	
nodular hyalinosis of more than one arteriole	7 (14.9)	31 (28.4)	
hyalinosis in the entire circumference	6 (12.8)	36 (33.0)	
Interstitial fibrosis and tubular atrophy (%)			
≤5.0	18 (38.3)	22 (20.2)	0.080
6.0-25.0	16 (34.0)	40 (36.7)	
25.0-50.0	8 (17.0)	34 (31.2)	
≥50.0	5 (10.6)	13 (11.9)	
Proteinuria (g/L)			
<1	3 (6.5)	12 (11.8)	<0.001
1-3.5	9 (19.6)	41 (40.2)	
3.6-10	12 (26.1)	40 (39.2)	
>10	22 (47.8)	9 (8.8)	
Erythrocytes in urine			
negative	16 (35.6)	48 (49.0)	0.134
positive	29 (64.4)	50 (51.0)	
Serum IgM (g/L)			
not performed	12 (26.7)	32 (32.3)	0.719
normal levels	29 (64.4)	55 (55.6)	
elevated levels	1 (2.2)	5 (5.1)	
decreased levels	3 (6.7)	7 (7.1)	
Therapy			
symptomatic	11 (24.4)	62 (60.8)	<0.001
immunosuppressive	34 (75.6)	40 (39.2)	
Anti-RAAS			
without anti-RAAS	2 (4.4)	8 (7.8)	0.451
anti-RAAS	43 (95.6)	94 (92.2)	

*Abbreviations: FSGS – focal segmental glomerulosclerosis, Ig – immunoglobulin, C – complement, RAAS – renin-angiotensin-aldosterone system.

†χ^2^ test.

‡Excluded patients without data for glomeruli analysis by electron microscopy.

### IgM deposition in primary and secondary FSGS

The FSGS group had a significantly higher prevalence of positive IgM deposition and positive C3 deposition than the control group (Table 1). Comparison among IgM+C3+, IgM+C3-, and IgM-C3- showed a significantly more prevalent positive IgM and C3 deposits in FSGS group.

According to the correlation coefficients between IgM and C3 deposition with other clinical and histological variables in primary and secondary FSGS, there were significant positive correlations between the IgM and C3 deposition and between IgM and mesangial deposits on EM in the secondary FSGS group, indicating that IgM deposition is associated with C3 deposition and EM mesangial deposits only in the secondary FSGS group (Table 4). There was a weak positivity on direct immunofluorescence for IgA in 3 patients with primary FSGS and 6 patients with secondary FSGS (Table 5). Direct immunofluorescence was also weakly positive for C1q in 3 patients with primary FSGS and 5 patients with secondary FSGS (Table 5). However, there was no significant correlation between IgA or C1q deposits and IgM and C3 deposition (Tables 4 and 5).

**Table 4:** Correlation coefficients (rho) between immunoglobulin (Ig) M and complement (C) 3 deposition with other clinical variables in primary and secondary focal segmental glomerulosclerosis (FSGS)

		Primary FSGS (n = 47)		Secondary FSGS (n = 109)	
Variable		IgM	C3	IgM	C3
IgM deposition	rho	1	0.065	1	0.372
	*P*		0.662		<0.001
C3 deposition	rho	0.065	1	0.372	1
	*P*	0.662		<0.001	
Mesangial deposits by electron microscopy	rho	0.162	-0.086	0.233	0.049
	*P*	0.276	0.566	0.015	0.613
C1q deposition	rho	0.153	-0.068	-0.092	0.001
	*P*	0.306	0.649	0.341	0.992
IgA deposition	rho	0.065	-0.068	0.147	0.080
	*P*	0.662	0.649	0.128	0.407
C3 serum	rho	-0.021	-0.243	-0.024	0.118
	*P*	0.887	0.099	0.808	0.222
C4 serum	rho	0.074	-0.238	-0.071	0.095
	*P*	0.622	0.107	0.464	0.327
IgM serum	rho	0.159	0.032	-0.028	-0.316
	*P*	0.352	0.853	0.814	0.008

**Table 5:** Differences in immunoglobulin (Ig) A and complement (C) 1q deposits regarding IgM and C3 deposition in primary and secondary focal segmental glomerulosclerosis (FSGS)

FSGS		No. (%) of patients		P*
		negative IgA	positive IgA	
Primary	IgM+, C3-	20 (45.5)	2 (66.7)	0.754
	IgM+, C3+	2 (4.5)	0 (0.0)	
	IgM-, C3-	22 (50.0)	1 (33.3)	
Secondary	IgM+, C3-	42 (40.8)	3 (50.0)	0.411
	IgM+, C3+	18 (17.5)	2 (33.3)	
	IgM-, C3-	43 (41.7)	1 (16.7)	

FSGS		negative C1q	positive C1q	
Primary	IgM+, C3-	20 (45.5)	2 (66.7)	0.754
	IgM+, C3+	2 (4.5)	0 (0.0)	
	IgM-, C3-	22 (50.0)	1 (33.3)	
Secondary	IgM+, C3-	43 (41.3)	2 (40.0)	0.481
	IgM+, C3+	20 (19.2)	0 (0.0)	
	IgM-, C3-	41 (39.4)	3 (60.0)	

*χ^2^ test.

### Treatment response

Although there was no significant difference in response to treatment between primary and secondary FSGS (complete or partial remission of the disease) when shown as a binary variable, the significantly higher prevalence of partial remission was found in secondary FSGS (*P* = 0.001).

Log-rank Mantel-Cox test of equality for the survival distributions of different intensity of IgM and C3 on IF for response to treatment (complete and partial remission) in the primary and secondary FSGS groups over time showed that patients in the primary FSGS group with positive C3 on IF reached response to treatment at an earlier time point than patients who had negative results for C3 on IF (*P* = 0.018).

To determine the risk factors for response to treatment (complete and partial remission) among patients with secondary FSGS, we used univariate and multivariate analysis. On univariate level, three significant predictors were found: urinary protein level, eGFR, and interstitial fibrosis and tubular atrophy (IFTA). In multivariate analysis, only lower concentration of urinary protein was found to be a significant predictor of disease remission (OR 0.48, 95% CI 0.24-0.99, *P* = 0.048). Male gender was only significant predictor of disease remission among patients with primary FSGS (OR 7.78, 95% CI 1.52-39.75, *P* = 0.014).

### Renal outcome

**Figure 2:**
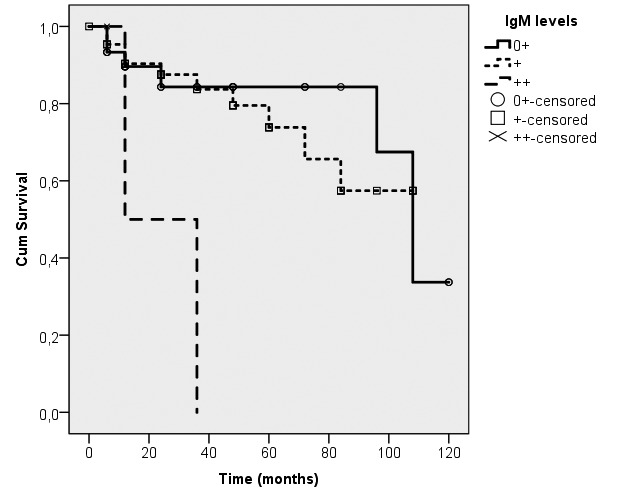
Kaplan-Meier survival curve showing that patients with secondary focal segmental glomerulosclerosis who had more immunoglobulin (Ig) M deposits on immunofluorescence reached the composite renal outcome at an earlier time point than patients with lower or negative IgM deposition (log-rank test; *P* = 0.022).

There were no significant differences between the primary and secondary FSGS groups in the composite renal outcome (permanent increase in serum creatinine by ≥50% or end-stage renal disease, a need for renal replacement therapy, dialysis or transplantation, or death) and number of re-biopsies. Patients in the secondary FSGS group who had more IgM deposits on IF reached the composite renal outcome at an earlier time point than patients with lower or negative IgM deposition (Figure 2, *P* = 0.022). In univariate analysis, urinary protein level and eGFR were shown as risk factors with a significant impact on the likelihood of progression of renal disease in patients with secondary FSGS (Table 6). When these predictors where analyzed in a multivariate regression model, higher levels of urinary protein increased the chance for progression of renal disease by 2.55 times, while higher eGFR lowered that chance. Female gender was the only significant predictor for progression of the renal disease among patients with primary FSGS.

**Table 6:** Risk factors for composite renal outcome (progression of the renal disease) among patients with secondary focal segmental glomerulosclerosis analyzed with binary logistic regression*

Composite renal outcome†	Univariate analysis		Multivariate analysis	
	HR (95% CI)	*P*	OR (95% CI)	*P*
Female gender	2.91 (0.76-11.10)	0.119		
Age (years; per 1 year)	1.01 (0.97-1.05)	0.697		
Urinary protein (per 1 g/24h)	2.30 (1.09-4.86)	0.029	2.55 (1.12-5.80)	0.026
eGFR (per 10 ml/min/1.73 m^2^)	0.97 (0.95-1.00)	0.019	0.98 (0.95-0.99)	0.031
IgM deposition: IgM- (reference)				
IgM+, C3-	1.05 (0.33-3.36)	0.933		
IgM+, C3+	1.31 (0.31-5.52)	0.709		
Intensity of IgM staining (per 1+)	1.46 (0.57-3.77)	0.429		
Intensity of C3 staining (per 1+)	1.57 (0.68-3.64)	0.290		
Mesangial deposits (per 1+)	1.36 (0.58-3.22)	0.482		
Interstitial fibrosis and tubular atrophy (%)	1.47 (0.81-2.66)	0.206		
Percentage of sclerosis (per 1%)	1.33 (0.11-15.94)	0.823		

*Abbreviations: HR – hazard ratio, CI – confidence interval, OR – odds ratio, eGFR – estimated glomerular filtration rate, Ig – immunoglobulin, C – complement.

†Composite renal outcome = permanent increase in serum creatinine by ≥50% or end-stage renal disease, a need for renal replacement therapy (dialysis or transplantation), or death.

## DISCUSSION

Our study showed that intraglomerular IgM and C3 deposits were frequently detected in both primary and secondary FSGS without the correlation with other relevant immunofluorescence parameters. Similarly to a previous study (18), our results imply that the detection of intraglomerular IgM and C3 deposits may serve as an additional diagnostic parameter for patients with faster progression of FSGS irrespective of the disease etiology. Our results further confirm the recent studies (17,18), challenging the older concept that trapped IgM is only an “innocent bystander”. In our study, the patients with FSGS had a significantly higher prevalence of positive IgM and C3 deposition than the control group. It is likely that the activation of the complement system may occur secondary to IgM natural antibody binding to neoantigens exposed upon glomerular stress irrespective of the nature of injurious stimulus further contributing to injury progression (17). IgM is generally regarded as an activator of classic complement pathway, but recent results suggest the activation of both classic and alternative pathways. Therefore, it is plausible that IgM activates classic pathway and then amplification of alternative pathway can be triggered (22).

Zhang et al (18) were the first to report the clinical significance of IgM and C3 deposition in patients with primary FSGS, but not in those with secondary FSGS. Also, they focused mainly on IgM and C3 deposits in sclerotic areas of glomeruli. We investigated the potential correlation between IgM and C3 diffuse mesangial deposits of non-sclerotic glomerular areas in kidney biopsy tissue of patients with primary and, for the first time in human pathology, with secondary FSGS. We found that the patients with secondary FSGS and diffuse mesangial IgM deposits had an earlier development of inferior renal outcomes compared with patients without mesangial IgM deposits. There were no significant correlations between the mesangial IgM deposits and clinical outcome in patients with primary FSGS, the finding that differs from the results of the study of Zhang et al (18). This could be explained by the small number of patients with primary FSGS in our study. In addition, a correlation between mesangial IgM and C3 deposits on IF in the secondary FSGS and between mesangial deposits detected by EM and IgM on IF were found. The previously reported study revealed that patients with primary FSGS and IgM deposition in glomeruli presented with higher level of serum IgM (18). In our study, there was no significant correlation between IgM/C3 deposits and serum IgM/C3 levels.

There is a historical controversy about the relevance of glomerular IgM deposits in the progression of nephrotic syndrome in pediatric and adult patients (3,23-29). In a recent large retrospective study, a high proportion of patients with simultaneously occurring intraglomerular IgM/C3 deposition failed to achieve remission and had refractory nephrotic syndrome (18). Contrary to these results, our study showed that patients with primary FSGS with positive C3 on IF reached response to treatment at an earlier time point than did patients who had negative results for C3. Primary FSGS cohort in our study was smaller than one in previously mentioned study. We are not sure if contradiction of our results relies on the cohort size difference or perhaps on the localization of analyzed IgM and C3 deposits. While previously study investigated mainly deposits in sclerotic areas, we focused on diffuse mesangial deposits. That may also establish the basis for new speculations on the significance of IgM/C3 found in sclerotic vs non sclerotic areas. Given our data showing a faster progression in secondary FSGS in patients with IgM/C3 deposits, we carefully suggest that ongoing intraglomerular immune response uncovers patients prone to inferior outcomes irrespective of the nature of glomerular disease. From the clinical point of view, targeting intraglomerular immune response beyond standard means of nephroprotection by RAAS blockade is worth considering for this particular group of patients. The notion on role of C3 in non-immune renal injury is not new. The classic experimental model of hypertensive injury in transgenic animals overexpressing human RAAS provides evidence that intraglomerular findings of complement components precede onset of proteinuria (30).

The limitations of our study are a small number of patients, especially those patients with primary FSGS, and retrospective design. Also, our study provides correlations and not a pathophysiologic proof of concept. However, we provide first human biopsy report in adult patients with secondary FSGS showing that two classical intraglomerular immunoflourescence parameters (IgM and C3) may contribute to the clarification of this very heterogeneous group of patients. Although interventions aimed at modulations of complement effector molecules are increasingly used in autoimmune renal diseases and transplantation, we believe that further analyses are necessary to draw conclusions about benefits of this kind of treatment in FSGS therapy (31).

In conclusion, this study showed a possible role of IgM deposition in secondary FSGS, indicating that IgM may serve as a novel predictor of disease progression. In our opinion, a further survey of the presence and possible pathogenic role of IgM in glomerulonephritis should be performed. Prospective studies with a larger patient cohort and therapy information are warranted to definitely define the role of IgM in patients with faster progression of FSGS.
